# Shared infections at the wildlife–livestock interface and their impact on public health, economy, and biodiversity

**DOI:** 10.1093/af/vfad067

**Published:** 2024-02-14

**Authors:** Dibesh Karmacharya, Gloria Herrero-García, Bibhu Luitel, Rajesh Rajbhandari, Ana Balseiro

**Affiliations:** One Health Division, Center for Molecular Dynamics Nepal, 44600 Kathmandu, Nepal; One Health Division, BIOVAC Nepal, 45210 Nala, Nepal; Department of Biological Sciences, University of Queensland, 4072 Brisbane, Australia; Departamento de Sanidad Animal, Facultad de Veterinaria, Universidad de León, 24071 León, Spain; One Health Division, BIOVAC Nepal, 45210 Nala, Nepal; One Health Division, Center for Molecular Dynamics Nepal, 44600 Kathmandu, Nepal; Departamento de Sanidad Animal, Facultad de Veterinaria, Universidad de León, 24071 León, Spain

**Keywords:** animal interactions, animal production, biosecurity measures, integrated control, shared animal infections

**Implications**

- Shared infections at the livestock–wildlife interface are of major concern to public health, economy, and biodiversity.
- Over 20% of global animal production losses are caused by animal diseases.
- Factors such as exponential growth in animal and human populations, rapid urbanization, evolving farming systems, increased interaction of livestock and wildlife, ecosystem changes, globalization of animal and animal product trade, and shifts in pathogen–host ecology contribute to the emergence of new disease interfaces.
- The spread of shared infectious diseases in any interface is influenced by multiple factors and can occur within communities with a complex structure, often with many hosts involved in infections transmission dynamics.
- One Health focused collaborative efforts involving multiple disciplines can be effective in improving health of animal, human, and environment in general.

## Introduction

Shared infections at the livestock–wildlife “interface” are commonly referred to as “spillover” or “cross-species transmitted” infections (Table 1). They underscore the interconnectedness of wildlife and livestock ecosystems and frequently impact multiple facets of public health, economy, and biodiversity. Lately, there has been a growing focus on these diseases, as evidenced by a multitude of publications on the subject (Wiethoelter et al. 2015). Understanding and, consequently, preventing these infections, is crucial for protecting animal health, safeguarding food security, and mitigating public health risks.

**Table 1.** Definition of terms

Term	Definition
Interface	Diverse direct and indirect interactions between livestock and wildlife, which can serve as possible pathways for the transmission of shared infections
Shared infections	Infections caused by transmissible pathogens that are sustained by at least one wild and one domestic animal host species
One health approach	Integrated, unifying approach that aims to sustainably balance and optimize the health of people, animals, and ecosystems
Direct contact	Coming into contact with the saliva, blood, urine, feces, or other body fluids of an infected animal, e.g., bites
Indirect contact	Coming into contact with areas where animals live and roam, or objects or surfaces that have been contaminated with pathogens
Emerging diseases	Outbreaks of previously unknown diseases; known diseases that are rapidly increasing in incidence or geographic range in the last two decades; or persistence of infectious diseases that cannot be controlled
Re-emerging diseases	Diseases that reappear after they have been on a significant decline
Biosecurity	Set of strategies that reduce risk of infectious diseases to an acceptable level in the facility and its immediate surroundings

Livestock and wildlife have coexisted, sharing and competing for resources in diverse ecosystems for millennia. However, as the human population continues to grow, resource sharing increasingly occurs at interfaces along the boundaries of heavily human-influenced environments (Vicente et al. 2021). Conversely, some changing dynamics, such as population increase of certain wildlife species, like the wild boar (*Sus scrofa*), have brought domestic and wild species into closer contact at these interconnecting interfaces, resulting in the spillover of infectious diseases (Santos et al. 2022).

Animal production, contributing to more than 40% of the total global agriculture output, plays a vital role in supporting the livelihoods of over >20% of the world’s population (FAO 2023). According to the World Health Organization (**WHO**) reports, over 20% of global animal production losses are caused by different animal diseases (WHO 2023). Additionally, infectious diseases in animals that have zoonotic potential pose significant risk to public and global health. Notably, the majority (>75%) of emerging infectious diseases (**EID**) in the last century were zoonotic in nature (Jones et al. 2008).

Gaining deeper insights into the epidemiology and ecology of shared infections is essential to prevent, curtail, and mitigate their impacts. A multifaceted approach, including wildlife disease surveillance, studies on disease ecology, and research on potential critical points of infections transmission at the interfaces, can prove effective in addressing many of these emerging and re-emerging diseases.

## Globally Relevant Shared Infections

**Figure 1.**
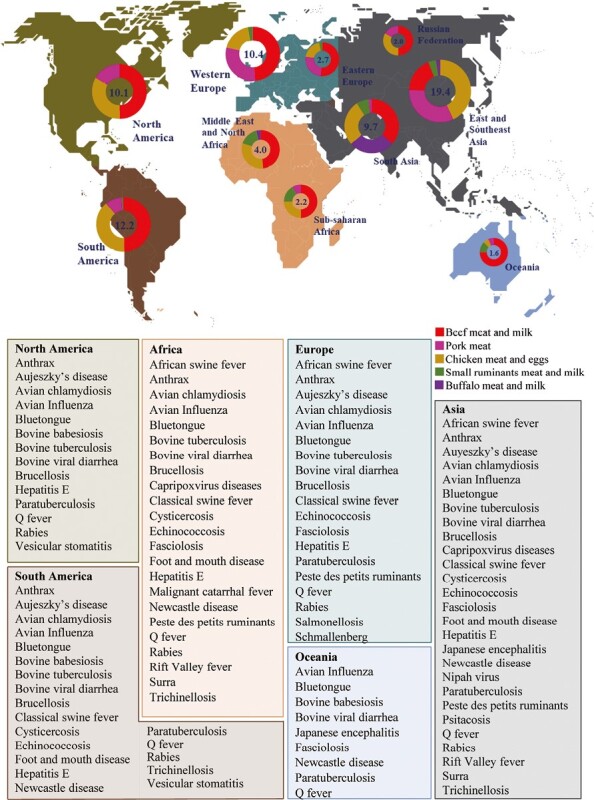
Notable shared infections in the livestock–wildlife interface categorized by continent and total animal production by regions. Protein quantities are indicated in millions of tons. The upper image has been modified based on data from a FAO source (https://www.fao.org/gleam/es/). The selection of shared infections has been adapted from Vicente et al. (2021), focusing on beef, pork, chicken, small ruminants, and buffalo species.

There are numerous important and relevant multihost shared infections worldwide, caused by bacteria, viruses, or parasites (Figure 1). Over the past 30 years, several infections and subsequent diseases at the livestock–wildlife interface have gained prominence due to their global impact on economy and public health. Some of the primary livestock species (i.e., poultry, pigs, bovines, small ruminants) shared diseases are: Avian Influenza (**AI**), salmonellosis, and New Castle disease in poultry; African swine fever (**ASF**), Aujeszky’s disease, and *Streptococcus suis* infection in pigs; tuberculosis (**TB**), brucellosis, and Rift Valley fever (**RVF**) in bovines; and peste des petits ruminants (**PPR**), foot and mouth disease (**FMD**), and bluetongue disease in small ruminants. These diseases have a global distribution and affect various livestock and wildlife species. Here, we highlight one representative infectious disease from each of the four animal production (food) species that are included in the single list of notifiable diseases of the World Organization for Animal Health (WOAH 2023).

AI, commonly known as bird flu, is caused by avian influenza Type A virus (family: *Orthomyxoviridae*, genus: *Alpha influenza virus*). AI affects both domestic and wild birds worldwide and spreads through direct contact with infected bird secretions or indirectly via contaminated feed, air, and water. Increased outbreak occurrences at the interface between animal production (poultry) and wild and migratory birds with extensive geographical reach have contributed to the global spread of the virus (Bessière et al. 2022). In 2022, 67 countries across five continents reported H5N1 high pathogenic avian influenza outbreaks in poultry and wild birds. In 2023, an additional 14 countries reported outbreaks, mainly in the Americas, as the disease continued its southward expansion (EFSA 2023). Moreover, AI’s spread has been exacerbated by the rapid globalization of human migration and trade, resulting in significant economic losses. For instance, AI-related losses in the US exceeded $3 billion in 2015 (Böckmann 2021). Overall, the epidemiology of AI has undergone significant changes in recent years, rendering it as one of the most challenging pathogens from a global health perspective. Its impact is particularly pronounced on bird biodiversity and the poultry industry’s economy (Bessière et al. 2022). Given the unprecedented spread of the H5N1 strain of AI virus among birds and mammals, the United Nations Health agencies (FAO, WHO, and WOAH) are urging countries to take some immediate preventive actions, such as strengthening biosecurity measures and promoting good hygiene practices, implementing comprehensive immunization of poultry, rapid detection and reporting animal outbreaks, and establishing an effective One Health based influenza surveillance system (EFSA 2023).

ASF is a highly contagious viral disease caused by a DNA virus (family: *Asfarviridae*, genus: *Asfivirus*) that affects both domestic pigs and wild boar. It is transmitted through direct and indirect contact, including through ticks. Outbreaks of ASF at the wildlife–livestock interface involving transmission between domestic and wild pigs have been commonly reported globally (Mugabi and Duffy 2023). ASF has traditionally been present in the African continent and had its first occurrence in the European Union in 2014. In August 2018, the virus was detected in the People’s Republic of China, and in 2021, the disease reappeared in the Americas (in the Dominican Republic and Haiti; WOAH 2022). It resulted in decrease in world pig meat production between 2018 and 2019 by 11 million tons. Pork is the second most widely consumed meat globally, having been recently overtaken by poultry meat (FAO 2023), and in recent years, ASF has emerged as one of the major challenges to the pork industry, leading to substantial economic losses, especially in Asian countries with recent continuous outbreaks reported since late 2018 (FAO 2022b). Estimated direct production loss due to ASF in the People’s Republic of China are expected to be around $55 billion to $130 billion (Weaver and Habib 2020). Lack of early detection system and containment strategies in many countries has helped spread of the disease. Proper biosecurity measures can be an effective universal preventive action to control ASF, since there is no treatment or vaccine for the disease (Urbano and Ferreira 2022).

TB is a chronic bacterial disease caused by the *Mycobacterium tuberculosis* complex—of which *M. tuberculosis* and *M. bovis* predominantly infect humans and livestock, respectively. TB is one of the most important global zoonotic diseases affecting humans, livestock and wildlife, and with the emerging challenges associated with the antimicrobial resistance to various antibiotics, TB has emerged as one of the greatest threats to the global health (Lee et al. 2022). The highest prevalence of bovine TB is in Africa and some regions of Asia, but the disease is globally present. Historically, TB affected humans and livestock; however, it has extended to wildlife as well (Cowie et al. 2016). In humans, over 80% of cases and deaths are in low- and middle-income countries, but TB occurs in every part of the world (TB is the 13th leading cause of death and the second leading infectious killer after COVID-19) (WHO 2023). In livestock, complete elimination of the disease is complicated by the persistent infection of wild animals. Between January 2017 and June 2018, 82 (44%) of the 188 countries and territories that notified the OIE/WOAH of their bovine TB status had been affected, indicating disease widespread distribution (Murai et al. 2019). Some of the interesting interfaces where TB transmission is known to spill-over within and between species are: human-Asian elephant (*Elephas maximus*) (captive and wild) in Nepal and India, European badger (*Meles meles*)-cattle in the UK, wild boar-cattle in the Iberian Peninsula, white-tailed deer (*Odocoileus virginianus*)-cattle in the United States, and African buffalo (*Syncerus caffer*)-cattle in South Africa (Cowie et al., 2016; Santos et al., 2022; Rajbhandari et al., 2023). There is inadequate data showing the scale of economic loss due to bovine TB in developing countries; however, in countries with a large livestock population such as Ethiopia, the loss is substantial (Azami and Zinsstag 2018).

PPR is caused by a RNA virus (family: *Paramyxoviridae*, genus: *Morbillivirus*) that affects 80% of the world’s sheep and goat population (Soula et al. 2018). PPR is highly contagious with a high mortality rate. Additionally, it also affects many of the wild ungulate species and pose both conservation and ecological threat. PPR is currently considered as one of the main animal transboundary infections that pose a direct threat to livestock production in many developing countries, particularly in western Africa and south Asia (Banyard et al. 2010), but also in Europe and the Middle East. The annual global economic impact of PPR has been estimated to be between US$1.4 and 2.1 billion (FAO 2022a). In 2015, the Peste des petits Ruminants Global Eradication Programme (PPR-**GEP**) was established and implemented by the FAO and the WOAH. Since then, strategic plans (vaccination, diagnosis, and surveillance and monitoring) have been implemented, and currently, 10 of the 67 countries with active or recent evidence of PPR infection have had no outbreaks between 2015 and 2019, supporting the positive impact of the control measures (FAO 2022a). The lack of knowledge regarding the role of wildlife in the epidemiology of this and other diseases at the wildlife–livestock interface hinders the implementation of necessary integrated disease control and management interventions for livestock.

## Factors Modifying Infection/Disease Interfaces

The emergence of new infection/disease interfaces is driven by several factors, including exponential growth in animal and human populations, rapid urbanization, evolving farming systems, closer integration between livestock and wildlife, encroachment into forests, shifts in ecosystems, globalization of animal and animal product trade, and changes in pathogen-host ecology. These factors also increase the likelihood of pathogen transmission between livestock and wildlife populations.

### Growing animal and human populations

**Figure 2.**
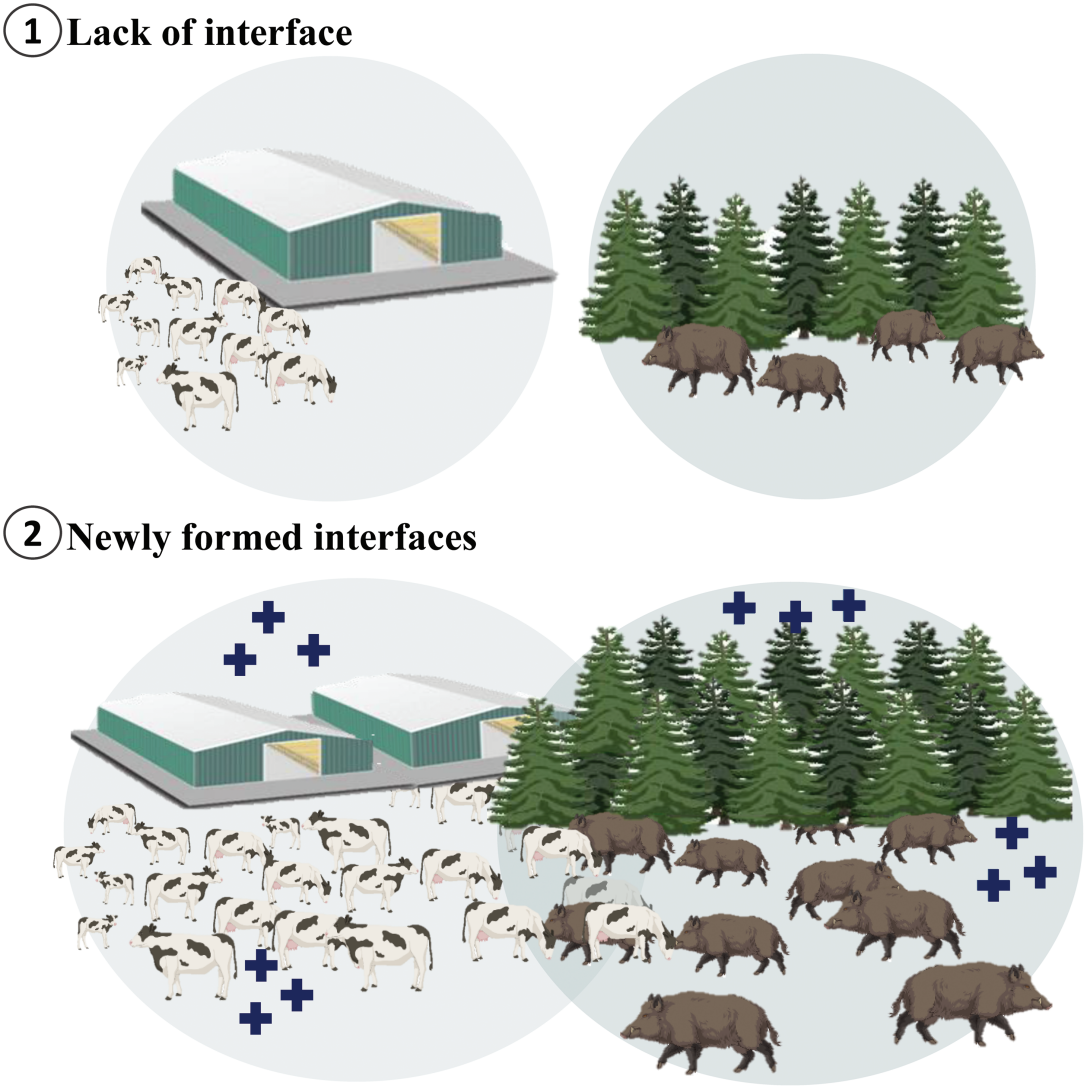
Newly formed interfaces (wildlife–livestock) resulting from the expansion of populations in livestock and wildlife species. Closer and open interfaces between animal production site and wildlife habitat mean higher likelihood of pathogen spillover and transmission. Transmission of African Swine Fever (ASF), caused by a DNA virus, between wild boar and domesticated (or farmed) pigs at a close livestock-wildlife interface is a good example of such scenario. A proper biosecurity measure in the animal production sites and creation of buffer zones between wildlife habitat and livestock sites can mitigate such pathogen (disease) spillover and transmission possibilities.

Currently, over a quarter of the world’s ice-free land, equivalent to over half of the agricultural land, is designated for livestock grazing, while roughly one-third of croplands are utilized for livestock feed production (FAO 2023). The global livestock population encompasses nearly 1.5 billion cattle, 2.2 billion small ruminants, over 35 billion chickens, and nearly 1 billion pigs. World meat [defined as the flesh of animals (excluding fish) used for food] production reached 337 million tons in 2020, up 45%, or 104 million tons compared with 2000, with chicken meat representing more than half the increase (FAO, 2022b). This increasing demand for production has resulted in habitat loss caused by the expansion of agriculture and livestock operations, forcing wild animals to seek food and shelter in proximity to animal production areas. Consequently, species are now experiencing higher levels of intermingling, leading to an increase in infection transmission (Figure 2). Simultaneously, wildlife populations, particularly ungulates, have been on the rise due to various factors such as declining legal hunting accompanied by a decrease in social acceptance (Gortázar et al. 2015). Elevated densities and expanded geographical distributions of susceptible hosts can also contribute to the proliferation of pathogens and the circulation of new variants and mutations (Baker et al. 2022). Traditional models for inter-species or vector-borne disease dynamics have typically operated on the assumption that the transmission rate is proportional to the local abundances of both the donor and recipient species (Becker et al. 2019). Therefore, promoting the safe coexistence of livestock and wildlife requires the integration of various approaches, including land use planning, pathogen control in production animals, implementation of biosecurity practices, and epidemiological surveillance.

### Biodiversity changes

Animal, human, and plant health are intricately linked and rely on the health of the ecosystems they inhabit. Their survival is intertwined. Unfortunately, global biodiversity, denoting the diversity and abundance of species within a specific ecosystem, is rapidly diminishing, surpassing any previous period in human history. This decline is primarily driven by human activities such as commercial pursuits, conversion of natural habitats for agriculture, urban sprawl, hunting, fishing, and the collection for local consumption or international trade (IUCN 2021).

The loss of biodiversity and the prevalence of shared infections among livestock and wildlife exhibit a positive correlation on a global scale (Pongsiri et al. 2009). This association is supported by the “dilution effect” hypothesis, which suggests that species-rich communities are more likely to restrict the spread of diseases, as pathogens encounter greater difficulty in encountering their competent hosts (hosts capable of transmitting infections to other susceptible hosts) (Barroso et al. 2023).

Furthermore, healthy ecosystems encompass a variety of species that play specific roles in regulating parasite and pathogen populations. When biodiversity is jeopardized, these regulatory mechanisms may falter, leading to diminished pathogen transmission dilution and heightened disease among both livestock and wildlife (Pongsiri et al. 2009). Conversely, the expansion of interfaces stemming from the degradation of natural habitats and the proximity of livestock to wildlife can intensify the likelihood of disease transmission across different species.

Addressing this challenge necessitates the implementation of measures conserving biodiversity and advocating for sustainable practices to mitigate the risk of disease spread. Preserving biodiversity in rural and natural environments entails ensuring the health of animals, maintaining diverse populations of livestock and wildlife species, and adopting appropriate stocking rates. By taking these steps, we can work toward safeguarding the delicate balance of ecosystems and mitigating the transmission of diseases among human, animal, and plant populations.

### Anthropogenic influence

The threat of emerging diseases is intricately linked to two ongoing environmental crises caused by human activity: globalization and climate change. These factors have the potential to modify the distribution of hosts, pathogens, and land use patterns. Urbanization, for instance, leads to increased concentration and interconnectedness of people and domestic animals, which can expedite the transmission of new infections. Likewise, globalization, driven by the integration of the global economy, has facilitated the spread of pathogens through the expansion of trade and travel (Wu 2017).

**Figure 3.**
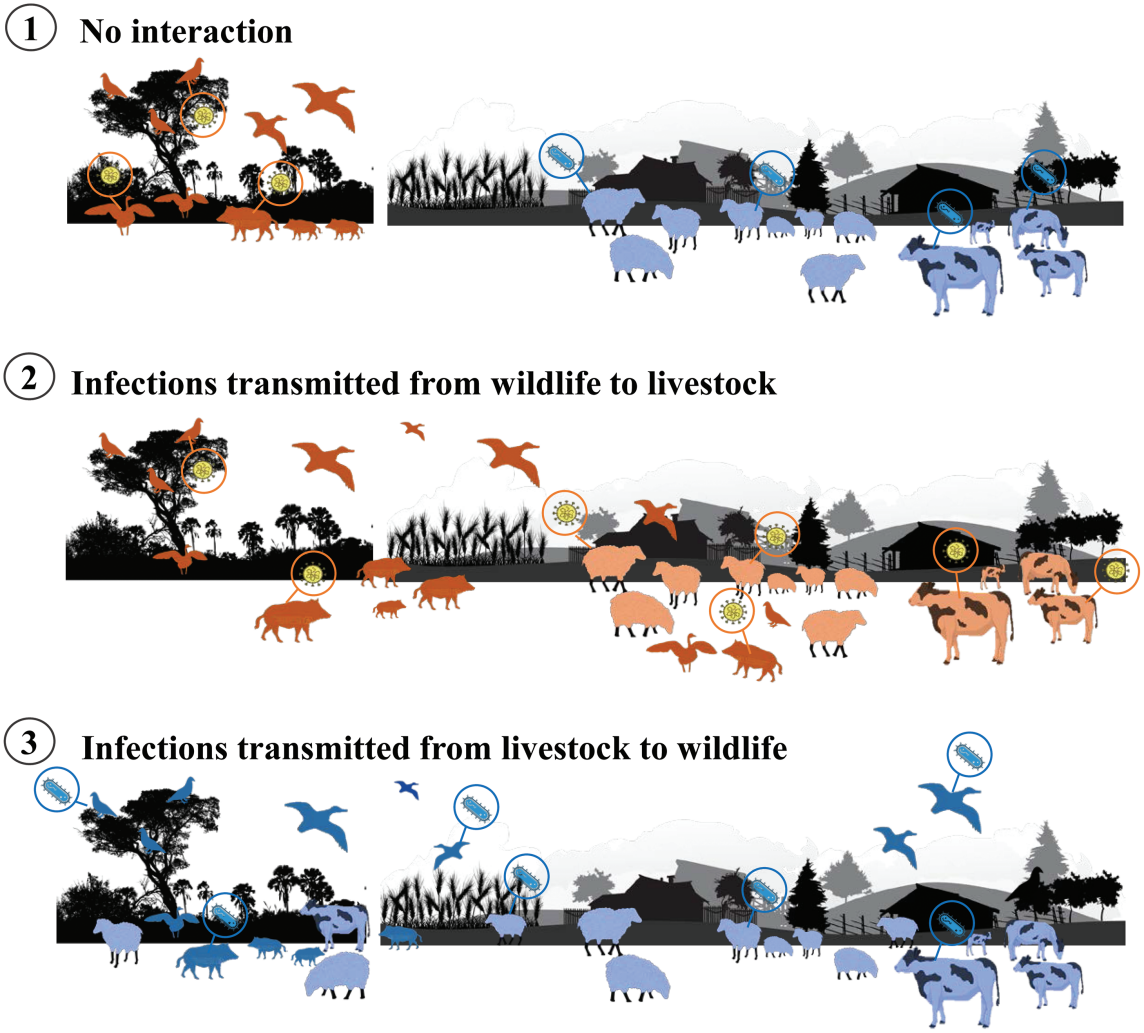
Increased connectivity between livestock and wildlife, and the introduction of pathogens to new hosts. In an ideal scenario, no interaction between livestock and wildlife would contain the spread of infections. However, this is a highly unlikely scenario considering ever growing human and livestock population. Once the interface between livestock and wildlife becomes thinner, pathogens that were circulating within wildlife and livestock ultimately starts spilling over and some of them (pathogens) gain higher transmission potential (especially RNA viruses) causing epidemics and even pandemics. With the growing emergence of Anti-Microbial Resistance (AMR) bacteria, mainly caused by haphazard and excessive use of antibiotics in animal productions, there is an increasing threat of AMR bacterial pathogens (such as *Mycobacterium tuberculosis* complex) infecting wild population of ungulates such as deer, wild boar, and rhinos.

As a result, human populations are moving into regions that were previously uninhabited, thereby increasing the likelihood of exposure to new wildlife disease reservoirs (Figure 3). The heightened connectivity also enables pathogens and their vectors to reach new hosts that may be phylogenetically or genetically similar to the original hosts but lack evolved defenses, consequently leading to more significant impacts on population and ecosystems (Shaw et al. 2020).

These intertwined factors of globalization and urbanization, coupled with the expansion of human populations into new areas, underscore the urgency of understanding and addressing the risks associated with emerging diseases. By recognizing the impact of human activity on pathogen transmission dynamics, we can work toward implementing proactive measures to mitigate the spread of diseases and protect both animal and human populations.

Additionally, intentional movements of animals driven by wildlife reintroduction and rewilding efforts, as well as the expansion of livestock trade, can significantly alter the dynamics of disease-mediated competition between species. An interesting example of this phenomenon is the expansion of gray wolves (*Canis lupus*) in certain regions of Spain, which has resulted in a decrease in TB among wild boar populations. The increased predation by wolves on sick boars has led to a compensatory reduction in disease-induced mortality (Tanner et al. 2019).

Climate change poses a major threat to global health and has been amplifying the risk of diseases in the livestock–wildlife interface. The changing climate conditions, including shifts in rainfall patterns, increased frequency of natural disasters, disrupted ecosystems, and alterations in landforms and vegetation patterns, create more favorable conditions for disease spread. These climate-induced changes have contributed to the emergence of new diseases (Baker et al. 2022). Furthermore, climate change can also impact the environmental reservoirs by influencing the populations of various bacteria, viruses, and fungi, by increasing, decreasing, or eliminating them. Epidemiologically, it is reasonable to expect that periods of global climate change will coincide with the emergence of new host–parasite relationships and the occurrence of outbreaks of emerging infectious diseases (Baker et al. 2022).

## Pathogen Transmission in Complex Maintenance Communities

Pathogen transmission within any interface is influenced by a multitude of factors. The spread of shared infections often occurs within complex maintenance communities, where multiple individuals play a role in perpetuating pathogen transmission. Several factors can impact this process, including disease ecology, characteristics of the pathogens involved, domestic and wildlife hosts, vectors, geographical scope, and socioeconomic contexts shaped by human activities. Consequently, each situation must be analyzed independently in order to comprehend and ultimately control it, necessitating an integrative perspective.

In systems characterized by multiple hosts and agents, various aspects related to population and community maintenance, the dilution effect, cross-species transmission, life cycles, and knowledge gaps must be addressed. For instance, Barroso et al. (2023) demonstrated that higher biodiversity and larger network sizes can actually reduce the number and prevalence of circulating pathogens, thereby potentially limiting transmission. However, this phenomenon is highly complex, with specific interactions between community hosts and environmental factors influencing the overall trend. In different epidemiological scenarios, such as TB in Mediterranean habitats, the prevalence of this multihost disease increased when wild boar and red deer interacted in such habitats (Santos et al. 2022).

## Control Measures at the Interface

**Figure 4.**
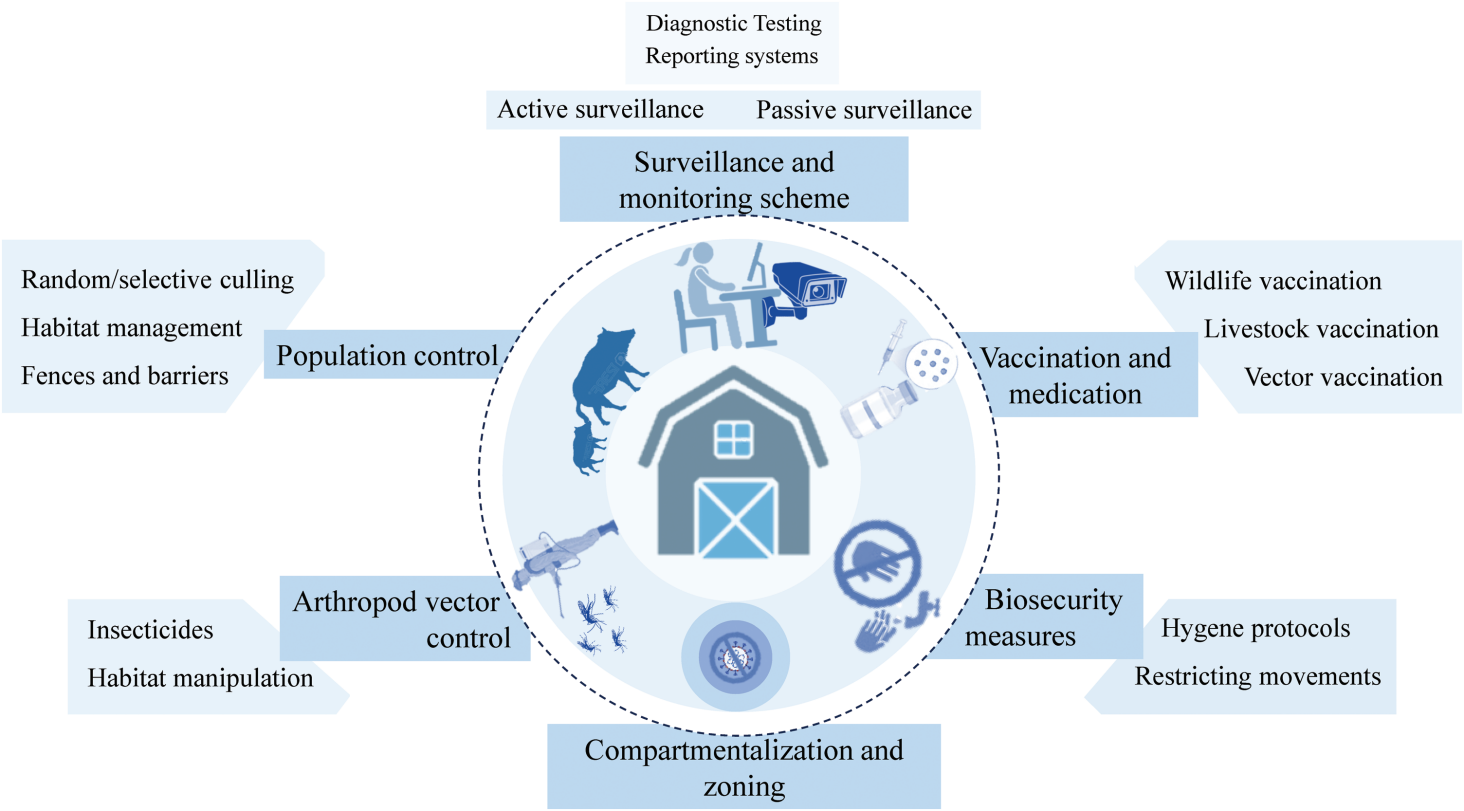
Combined control measures to prevent or reduce shared infections at the livestock-wildlife interface. A One Health-based approach of surveilling and preventing the pathogens from infecting livestock and wildlife is the most effective way of controlling infections at wildlife–livestock interface. This entails implementation of a comprehensive biosecurity measures at the animal production sites.

Control measures at the wildlife–livestock interface are typically implemented to minimize infections transmission and address conflicts among stakeholders, such as farmers, hunters, and official veterinarians. Taking a One Health perspective, which considers the interconnectedness of the environment, animals, and humans, is crucial for effective disease control in this interface. Control measures encompass various strategies, including active and passive sanitary surveillance of wildlife and livestock to promptly detect emerging or re-emerging diseases, population density management in cases of rapid growth, vaccination programs, vector control, and implementing biosecurity measures to prevent contact between domestic animals and wildlife (Figure 4). It is important to recognize that control measures may differ depending on the specific wildlife and livestock species involved, the pathogens at play, as well as the local context and regulations.

Regular infection and disease surveillance of both wildlife and livestock populations is essential for early pathogen detection and swift response. This monitoring can involve active surveillance programs, diagnostic testing, and robust reporting systems (Gortázar et al. 2015; Barroso et al. 2023). However, surveillance efforts in the wildlife–livestock interface pose significant challenges, often due to the limited availability of ecological and epidemiological data for wildlife species and technical difficulties associated with noninvasive methodologies (Miller et al. 2022). In regions with financial constraints, passive surveillance approaches, such as collecting and analyzing wildlife carcasses found in natural habitats, may be the only available option.

Controlling wildlife diseases often necessitates interventions in natural ecosystems, such as the implementation of fences and barriers, habitat management, and random or selective culling. However, these measures often give rise to controversies and debates (Glidden et al. 2021). In light of this, alternative strategies are being explored to better understand ecosystem-level processes, variability, and dynamics (Bohmann et al. 2014). One such approach involves investigating host-pathogen linkages by utilizing genetic material obtained from environmental samples. This approach offers several advantages, including its efficiency, non-invasiveness, and ease of standardization, and is currently under study.

Vaccination of domestic livestock against specific diseases plays a crucial role in reducing the risk of transmission to and from wildlife (Balseiro et al. 2020). Vaccines not only protect the vaccinated individuals but also provide community-wide benefits. By immunizing a large number of individuals against a particular pathogen, the exposure of non-vaccinated individuals to the pathogen is reduced, disrupting or minimizing the chain of disease transmission. This concept, known as “herd immunity,” is achieved when a significant portion of the population is immunized against a specific pathogen (Ellwanger et al. 2021). However, the implementation of vaccination programs can be complex, particularly in low-income regions.

Animal vectors, such as ticks or mosquitoes, are responsible for transmitting over 17% of all infectious diseases worldwide (WHO 2023). Vector control strategies need to be tailored to the specific requirements of each location, country, and epidemiological conditions. These strategies may involve the use of insecticides, predator species, habitat manipulation, house improvements, release of genetically modified mosquitoes, and other approaches (Wilson et al. 2020). Vaccines, for example, could serve as an effective and environmentally friendly option to control vector infestations and pathogen infection (de la Fuente et al. 2016). As mentioned in “Factors modifying infection/disease interfaces” section, climate change poses a major threat to disease appearance. In this framework, climate change can facilitate the expansion of disease vectors, such as mosquitoes and ticks, into new geographic areas. Warmer temperatures and altered precipitation patterns can create more favorable conditions for them to survive and reproduce. As a result, livestock and wildlife that were previously not exposed to certain diseases may become susceptible, increasing the risk of disease spill over. Changes in rainfall patterns can also affect the survival and persistence of pathogens in the environment (Baker et al. 2022).

Implementing biosecurity measures on livestock farms is crucial for preventing the introduction and spread of diseases. Research has shown that most domestic–wildlife interactions are indirect (Cowie et al. 2016). Enhancing biosecurity in the interface includes measures, such as restricting wildlife access, implementing hygiene protocols, and managing livestock movements. Modifying habitats to reduce interactions between wildlife and livestock, such as using fences to separate wildlife and livestock areas and minimizing indirect contact, can also be effective. While the risks associated with interactions in intensive production systems are well understood, there is a need for detailed, specific, and systematic protocols to assess biosecurity in extensive production systems (Martínez-Guijosa 2021).

Some measures can be expensive and difficult to achieve; however, zoning, i.e., identify specific geographical areas with defined status regarding a particular disease to apply different regulations and interventions, and compartmentalization, i.e., procedure used to define and manage an animal subpopulation of specified animal health status, might facilitate and reduce the cost of implementing control measures. These are used as prevention, management or eradication of animal disease and can provide the basis for exporting animals and their products after an outbreak.

In addition, control measures should not be used as standalone tools and should be accompanied by education and awareness among farmers, livestock owners, and local communities, promoting better understanding and cooperation.

## Conclusion

Shared infectious diseases at the livestock–wildlife interface have significant impacts on animal production and biodiversity. With the expected growth of selected wildlife populations, such as wild boar and synanthropic bird species, and global livestock production and trade, these diseases are likely to emerge, re-emerge and cause major epidemics in the future. Additionally, with the changing climatic conditions, the disease spill over and transmission dynamics have been changing as well, triggering more outbreaks at all different interfaces, mostly viral diseases, within all production groups. Therefore, it is essential to adopt a collaborative approach within the framework of One Health to safeguard the health of livestock, wildlife, humans, and the environment. To tackle the emerging and re-emerging diseases at wildlife–livestock interfaces, it is crucial to establish an enhanced disease surveillance system, implement effective risk mitigation and management strategies, and foster coordination and collaboration among national, regional, and global government and non-government agencies and stakeholders.

## About the Authors

**Dibesh Karmacharya** has a Conservation Biology degree from the Wayne State College (USA) and a PhD on Conservation and Microbiome Genetics from the Griffith University (Australia). He worked on transgenic animal models at Caliper Lifesciences (USA) as a research scientist. He promoted Genomics and Proteomics technology platforms for GE Healthcare Lifesciences in the US and Canada. He is the founder/chair of the Center for Molecular Dynamics Nepal, a wildlife genetics and clinical epidemiology research center. He also founded Intrepid Nepal Pvt. Ltd., a molecular diagnostics-based Biotechnology Company, and Intrepid Cancer Diagnostics, a leading cancer diagnostic laboratory. He is Founder/Director of BIOVAC Nepal- a vaccine research, development, and manufacturing company. He leads several innovative research on environment, health, and agro sectors. He has been developing “Early Warning System” for infectious diseases through innovative One Health-based genomics environmental surveillance.

**Gloria Herrero-García** graduated in Veterinary Medicine in the University of Zaragoza (Spain). She completed her internships at the Animal Production and Health Unit, specifically in the Brucellosis Group, at the Agrifood Research and Technology Centre of Aragon (CITA). She then obtained a MSc degree in Basic and Applied Investigation on Hunting Resources at the University of Castilla La—Mancha (Spain). During this time, she worked with the Research Group in Health and Biotechnology (SaBio) at the Institute for Game and Wildlife Research (IREC). Currently, Gloria is a predoctoral student at the Animal Health Department of the University of León (Spain). Her research focuses on tuberculosis, the livestock-wildlife interface and the implementation of biosecurity measures in multihost, multiagent systems.

**Bibhu Luitel** from Kathmandu (Nepal) possesses an MA in South Asian Studies from Pondicherry University (India). Her intellectual pursuits center around comprehending conservation efforts, ecological variations, environmental concerns, and political systems through a feminist and anticaste viewpoint. With a diverse professional background, Bibhu has collaborated with numerous organizations and served as a consultant for different entities. She specializes in conducting threat analysis on research related to biodiversity, conservation, and various aspects of human–wildlife interactions. She also develops GESI plans, by taking into account the diverse needs, perspectives, and experiences of all members of society, including marginalized and vulnerable groups such as women, ethnic minorities, and persons with disabilities.

**Rajesh Man Rajbhandari** is a seasoned professional with over a decade of experience in the fields of wildlife, biodiversity, public health, and social research. Rajesh is a Program Director of the Center for Molecular Dynamics Nepal (Nepal), and he specializes in studying wildlife diseases, environmental health, and sociobehavioral compositions. He is also involved in monitoring and evaluation of operational research. Rajesh’s expertise has been instrumental in research collaborations with esteemed international organizations including WHO, USAID, UCDAVIS, OXFORD, Emory University, and the Gates Foundation. His valuable research findings have been published in several peer-reviewed journals, showcasing his extensive knowledge in One Health.

**Ana Balseiro** is a veterinarian pathologist who graduated from the University of Leon (Spain) with a degree in Veterinary Medicine. She completed her PhD and dedicated 20 years of her career to be a researcher at the Animal Health Department of SERIDA (Principality of Asturias, Spain). She then joined the Animal Health Department of the University of Leon, where she currently serves as a researcher and lecturer of cytology, histology, and pathology. Throughout her career, Ana has focused on studying various infectious diseases that are shared between domestic animals and wildlife. She has led several international and national research projects. Her work aims to control these diseases through diverse approaches, including pathology and epidemiology of the diseases, development of new diagnostic techniques and vaccines, and biosafety measures.

## References

[CIT0001] Azami HY , Zinsstag J. 2018. Economics of bovine tuberculosis: a One Health issue. In: Chambers, M, Gordon S, Olea-Popelka F, Barrow P, editors. Bovine tuberculosis. Wallingford, Oxfordshire, England: CABI Books; 31–42. Chapter 3. doi:10.1079/9781786391520.0031

[CIT0002] Baker RE , Mahmud AS, Miller IF, Rajeev M, Rasambainarivo F, Rice BL, Takahashi S, Tatem AJ, Wagner CE, Wang L, et al. 2022. Infectious disease in an era of global change. Nature Rev. Microbiol. 20(4):193–205. doi:10.1038/s41579-021-00639-z 34646006 PMC8513385

[CIT0003] Balseiro A , Thomas J, Gortázar C, Risalde MA. 2020. Development and challenges in animal tuberculosis vaccination. Pathogens 9(6):472–428. doi:10.3390/pathogens9060472 32549360 PMC7350370

[CIT0004] Banyard AC , Parida S, Batten C, Oura C, Kwiatek O, Libeau G. 2010. Global distribution of peste des petits ruminants virus and prospects for improved diagnosis and control. J. General Virol. 91(12):2885–2897. doi:10.1099/vir.0.025841-0 20844089

[CIT0005] Barroso P , Relimpio D, Zearra JA, Cerón JJ, Palencia P, Cardoso B, Ferreras E, Escobar M, Cáceres G, López-Olvera JR, et al. 2023. Using integrated wildlife monitoring to prevent future pandemics through one health approach. One Health 16(100479):1–9. doi:10.1016/j.onehlt.2022.100479 PMC9806683 36600947

[CIT0006] Becker DJ , Washburne AD, Faust CL, Mordecai EA, Plowright RK. 2019. The problem of scale in the prediction and management of pathogen spillover. Phil. Transac. Royal Soc. 374(20190224):1–9. doi:10.1098/rstb.2019.0224 PMC6711304 31401958

[CIT0007] Bessière P , Dupré G, Volmer R. 2022. Controlling the emergence of highly pathogenic avian influenza viruses: one of the challenges of the 21st century? Virologie 26(5):343–354. doi:10.1684/vir.2022.0971 36413120

[CIT0008] Böckmann HC. 2021. Avian influenza outbreaks in Germany and the USA - comparison and analysis Wissenschaft und Innovation für Nachhaltige Geflügelwirtschaft (WING). Germany: Vechta.

[CIT0009] Bohmann K , Evans A, Gilbert MTP, Carvalho GR, Creer S, Knapp M, Yu DW, De Bruyn M. 2014. Environmental DNA for wildlife biology and biodiversity monitoring. Trends Ecol. Evol. 29(6):358–367. doi:10.1016/j.tree.2014.04.003 24821515

[CIT0010] Cowie CE , Hutchings MR, Barasona JA, Gortázar C, Vicente J, White PCL. 2016. Interactions between four species in a complex wildlife: livestock disease community: implications for Mycobacterium bovis maintenance and transmission. Eur. J. Wildlife Res. 62:51–64. doi:10.1007/s10344-015-0973-x

[CIT0011] de la Fuente J , Kopáček P, Lew‐Tabor A, Maritz‐Olivier C. 2016. Strategies for new and improved vaccines against ticks and tick‐borne diseases. Parasite Immunol. 38(12):754–769. doi:10.1111/pim.12339 27203187

[CIT0012] Ellwanger JH , Veiga ABG, Kaminski VDL, Valverde-Villegas JM, Freitas AWQ, Chies JAB. 2021. Control and prevention of infectious diseases from a One Health perspective. Genetics Mol. Biol. 44:1–23. doi:10.1590/1678-4685-GMB-2020-0256 PMC7856630 33533395

[CIT0013] EFSA (European Food Safety Authority), ECDC (European Centre for Disease Prevention and Control), EURL (European Reference Laboratory for Avian Influenza), Adlhoch C, Fusaro A, Gonzales JL, Kuiken T, Marangon S, Stahl K, Niqueux É, Staubach C, Terregino C, Mirinaviciute G, et al. 2023. Scientific report: Avian influenza overview December 2022–March 2023. EFSA J. 21(3):7917, 1-43. doi:10.2903/j.efsa.2023.7917 PMC10025949 36949860

[CIT0014] Food and Agriculture Organization of the United Nations (FAO). 2022a. Progress Toward the Eradication of Peste des Petits Ruminants. COAG:LI/2022/6. Rome. [accessed 25 August 2023]. https://www.fao.org/3/ni008en/ni008en.pdf

[CIT0015] Food and Agriculture Organization of the United Nations (FAO). 2022b. World Food and Agriculture – Statistical Yearbook 2022. Rome. [accessed 25 August 2023]. doi:10.4060/cc2211en

[CIT0016] Food and Agriculture Organization of the United Nations (FAO). 2023. Food Outlook – Biannual Report on Global Food Markets. Food Outlook, June 2023. Rome. [accessed 1 September 2023]. doi:10.4060/cc3020en

[CIT0017] Glidden CK , Nova N, Kain MP, Lagerstrom KM, Skinner EB, Mandle L, Sokolow SH, Plowright RK, Dirzo R, De Leo GA, et al. 2021. Human-mediated impacts on biodiversity and the consequences for zoonotic disease spillover. Curr. Biol. 31(19):R1342–R1361. doi:10.1016/j.cub.2021.08.070 34637744 PMC9255562

[CIT0018] Gortázar C , Diez-Delgado I, Barasona JA, Vicente J, de La Fuente J, Boadella M. 2015. The wild side of disease control at the wildlife-livestock-human interface: a review. Front. Vet. Sci. 1(27):1–12. doi:10.3389/fvets.2014.00027 PMC4668863 26664926

[CIT0019] International Union for Conservation of Nature (IUCN). 2021. Measuring Contributions Towards Biodiversity Targets [accessed 19 May 2023]. https://www.iucn.org/sites/default/files/2022-04/star_issues_brief_final_v.pdf

[CIT0020] Jones KE , Patel NG, Levy MA, Storeygard A, Balk D, Gittleman JL, Daszak P. 2008. Global trends in emerging infectious diseases. Nature 451(7181):990–993. doi:10.1038/nature06536 18288193 PMC5960580

[CIT0021] Lee S , Fan P, Liu T, Yang A, Boughton RK, Pepin KM, Miller RS, Jeong KC. 2022. Transmission of antibiotic resistance at the wildlife-livestock interface. Commun. Biol. 5(585):1–12. doi:10.1038/s42003-022-03520-8 35705693 PMC9200806

[CIT0022] Martínez-Guijosa J , Lima-Barbero JF, Acevedo P, Cano-Terriza D, Jiménez-Ruiz S, Barasona J Á, Boadella M, García-Bocanegra I, Gortázar C, Vicente J. 2021. Description and implementation of an On-farm wildlife risk mitigation protocol at the wildlife-livestock interface: tuberculosis in mediterranean environments. Prev. Vet. Med. 191(105346):1–10. doi:10.1016/j.prevetmed.2021.105346 33895501

[CIT0023] Miller RS , Bevins SN, Cook G, Free R, Pepin KM, Gidlewski T, Brown VR. 2022. Adaptive risk‐based targeted surveillance for foreign animal diseases at the wildlife‐livestock interface. Transboundary Emerg. Dis. 69(5):e2329–e2340. doi:10.1111/tbed.14576 PMC9790623 35490290

[CIT0024] Mugabi F , Duffy KJ. 2023. Epidemiological drivers and control strategies for African swine fever transmission cycles at a wildlife-livestock interface. Ecol. Model. 481(110344):110344–110349. doi:10.1016/j.ecolmodel.2023.110344

[CIT0025] Murai K , Tizzani P, Awada L, Mur L, Mapitse NJ, Caceres P. 2019. Panorama 2019-1: bovine tuberculosis: global distribution and implementation status of prevention and control measures according to WAHIS data. OIE Bullet. 1(3):1–5. doi:10.20506/bull.2019.1.2912

[CIT0026] Pongsiri MJ , Roman J, Ezenwa VO, Goldberg TL, Koren HS, Newbold SC, Ostfeld RS, Pattanayak SK, Salkeld DJ. 2009. Biodiversity loss affects global disease ecology. Bioscience 59(11):945–954. doi:10.1525/bio.2009.59.11.6.

[CIT0027] Rajbhandari RM , Napit R, Manandhar P, Raut R, Gurung A, Poudel A, Shrestha N, Sadaula A, Karmacharya D, Gortázar C, et al. 2023. Phylogenomic analysis supports Mycobacterium tuberculosis transmission between humans and elephants. Front. Vet. Sci. 10:1–8. doi:10.3389/fvets.2023.1133823. PMC10250650 37303725

[CIT0028] Santos N , Ferreras-Colino EEF, Arnal MC, de Luco DF, Sevilla I, Garrido J.M, Fonseca E, Valente AM, Balseiro A, Queirós J, et al. 2022. Complementary roles of wild boar and red deer to animal tuberculosis maintenance in multi-host communities. Epidemics 41(100633):1–11. doi:10.1016/j.epidem.2022.100633 36174428

[CIT0029] Shaw LP , Wang AD, Dylus D, Meier M, Pogacnik G, Dessimoz C, Balloux F. 2020. The phylogenetic range of bacterial and viral pathogens of vertebrates. Mol. Ecol. 29(17):3361–3379. doi:10.1111/mec.15463. 32390272

[CIT0030] Soula JJ. 2018. Panorama 2018-2. The Peste des Petits ruminants global research and expertise network (PPR–GREN). OIE Bulletin 2:1–87. doi:10.20506/bull.2018.2.2861

[CIT0031] Tanner E , White A, Acevedo P, Balseiro A, Marcos J, Gortázar C. 2019. Wolves contribute to disease control in a multi-host system. Sci. Rep. 9(8940): 1–12. doi:10.1038/s41598-019-44148-9 31138835 PMC6538665

[CIT0032] Urbano AC , Ferreira F. 2022. African swine fever control and prevention: an update on vaccine development. Emerg. Microb. Infect. 11(1):2021–2033. doi:10.1080/22221751.2022.2108342 PMC9423837 35912875

[CIT0033] Vicente J , Vercauteren KC, Gortázar C. 2021. Diseases at the wildlife-livestock interface: research and perspectives in a changing world. 3rd ed. Switzerland: Springer Nature.

[CIT0034] Weaver TR , Habib N. 2020. Evaluating losses associated with African swine fever in the People’s Republic of China and neighboring countries. Asian Develop. Bank 27:1–41. doi:10.22617/WPS200263-2

[CIT0035] Wiethoelter AK , Beltrán-Alcrudo D, Kock R, Mor SM. 2015. Global trends in infectious diseases at the wildlife–livestock interface. Proc Natl Acad Sci. 112(31):9662–9667. doi:10.1073/pnas.1422741112 26195733 PMC4534210

[CIT0036] Wilson AL , Courtenay O, Kelly-Hope LA, Scott TW, Takken W, Torr SJ, Lindsay SW. 2020. The importance of vector control for the control and elimination of vector-borne diseases. PLOS Neglect Trop. Dis. 14(1):1–31. doi:10.1371/journal.pntd.0007831 PMC6964823 31945061

[CIT0037] World Health Organization (WHO) 2023. World health statistics 2023: monitoring health for the SDGs, sustainable development goals. Geneva: Licence:CC BY-NC-SA 3.0 IGO.

[CIT0038] World Organization for Animal Health (WOAH). 2022. Disease Data Collection [accessed 3 September 2023]. https://www.woah.org/en/what-we-do/animal-health-and-welfare/disease-data-collection/

[CIT0039] World Organization for Animal Health (WOAH). 2023. Animal Diseases [accessed 3 May 2023]. https://www.woah.org/en/what-we-do/animal-health-and-welfare/animal-diseases/?_search=avian%20influenza

[CIT0040] Wu T , Perrings C, Kinzig A, Collins JP, Minteer BA, Daszak P. 2017. Economic growth, urbanization, globalization, and the risks of emerging infectious diseases in China: a review. Ambio 46:18–29. doi:10.1007/s13280-016-0809-2 27492678 PMC5226902

